# Effect of chronic khat (*Catha edulis,* Forsk) use on outcome of *Plasmodium berghei* ANKA infection in Swiss albino mice

**DOI:** 10.1186/s12879-015-0911-2

**Published:** 2015-04-02

**Authors:** Tsige Ketema, Moti Yohannes, Esayas Alemayehu, Argaw Ambelu

**Affiliations:** Department of Environmental Health Sciences and Technology, College of Health Sciences, Jimma University, Jimma, Ethiopia; School of Civil and Environmental Engineering, Jimma University Institute of Technology, Jimma, Ethiopia; Department of Biology, College of Natural Sciences, Jimma University, Jimma, Ethiopia; Department of Microbiology and Veterinary Public Health, College of Agriculture and Veterinary Medicine, Jimma University, Jimma, Ethiopia

**Keywords:** Cerebral malaria, Leucopenia, Pulmonary edema, *P. berghei*, Thrombocytopenia

## Abstract

**Background:**

The objective of this study was to explore effects of khat (*Catha edulis*) on outcome of rodent malaria infection and its anti-plasmodial activities on *Plasmodium berghei* ANKA (*PbA*).

**Methods:**

Female Swiss albino mice were orally treated with crude khat (*Catha edulis*) extracts (100, 200 and 300 mg/kg) on a daily basis for 4 weeks prior to *PbA* infection. Physical, clinical, hematological, biochemical and histo-pathological features of the mice were assessed. In addition, *in vivo* anti-plasmodial activities of khat were evaluated.

**Results:**

The finding of this study showed that khat use was strongly associated with increment of levels of liver and kidney biomarkers, leucopenia, severe anemia, rise in level of inflammation biomarkers: C-reactive protein (CRP), uric acid (UA), increased monocyte-lymphocyte count ratio (MLCR), manifestation of cerebral malaria symptoms such as ataxia, paralysis and deviation of the head but with no pulmonary edema. Significantly lower level of parasitemia (P < 0.05), rectal temperature, but, high level of hemoglobin were observed at the early stage of the *PbA* infection in khat treated mice than the control. With extension of the treatment period, however, drastic increments were observed in parasite load and rectal temperature although there was reduction in hemoglobin (Hb) level. Moreover, khat showed poor anti-plasmodial activity with <10% parasite suppression activity and lack protection against major malaria symptoms. The significant reduction (P < 0.01) of hematological parameters during *PbA* infection strengthen the notion that hematological parameters could be good predictors of severe malaria complications in human.

**Conclusions:**

In mice model treated with khat prior to infection with the rodent malaria parasite, khat was found to worsen manifestation of most malaria complications. Furthermore, the same plant showed poor *in vivo* anti-plasmodial activity and protection against major malaria symptoms.

## Background

Khat (*Catha edulis*, Forsk), a natural stimulant, is chewed by millions of people in Yemen, Somalia, Ethiopia, Djibouti and Kenya [1]. Nowadays, the habit of khat chewing is spreading to Europe, North America, Australia and Canada [2-4]. Besides its stimulating role, the processed leaves and roots of the plant are used for treatment of various ailments including influenza, cough, asthma, gonorrhea, vomiting and headache [1,5]. In addition, some endogenous people of East Africa and Meru tribe of Kenya use khat to treat malaria [1,5-7]. Similarly, some people in Yemen use khat for treatment of obesity, suppression of appetite and to alleviate their headaches by inhaling the fumes of burning khat leaves [8].

Khat contains compounds such as alkaloids, terpenoids, flavonoids, sterols, glycosides, tannins, amino acids, vitamins and minerals [9]. However, the stimulating effect of khat is mainly associated with its alkaloids content, cathinone and to a lesser extent cathine and norephedrine [10]. Cathinone is an intermediate metabolite in the biosynthesis of cathine which is found mainly in young fresh leaves of khat plant [10]. Khat, when chewed, is rapidly absorbed after oral administration, and it reaches peak plasma levels within 1.5-3.5 hrs after the onset of chewing of khat [11,12]. Cathinone is relatively unstable and decomposes within few days of picking, or if the leaf is dried, it changes into cathine and norephedrine [13].

Several reports showed that khat could cause different health problems including increased susceptibility to cognitive impairment, cardiovascular disorders, stomach ulcer and increment in adrenocorticotrophic hormone levels. It also causes urine retention and gall bladder motility, gastro-intestinal tract constipation and hemorrhage due to tannin and norpesudoephedrine content of the plant [1,14-16]. Despite its publicized health consequences, the practice of khat use is increasing globally and becoming an everyday drug for most people of East Africa, including Ethiopia [16].

The co-existence of khat chewing habit and malaria endemicity in some parts of Ethiopia and people’s perception on medicinal value of the plant against malaria infection initiate an assumption that khat might have an effect on the outcome of malaria infection. This prompted the current study aimed to explore *in vivo* effect of crude khat extract on the outcome of malaria infection and its anti-plasmodial activity under controlled conditions of potential confounding factors using Swiss albino mice.

## Methods

### Experimental animal

In this study female Swiss albino mice, aged 7–8 weeks, weighing 25-30 g, obtained from Ethiopian Public Health Institute (EPHI), Addis Ababa, were used. The mice were housed in transparent plastic cage having SS sipper 250 ml water bottle. Wood shaving was used as bedding and it was replaced every morning after the cage was cleaned and disinfected with 70% alcohol. The animals were kept under unlimited access to commercial pellet food and fresh tap water *ad libitum*. The experimental room had 12/12 hrs light/dark cycles, 55% ± 5.7 relative humidity and 21°C mean temperature.

### Collection and extraction of khat

Fresh khat leaves were purchased from a local market. Shoot and leaves were carefully picked and chopped into small pieces (~0.5 cm^2^) using grinding machine and mixed in methanol (analytical grade). For 100 g of the plant, 300 ml of methanol was used. Methanol was chosen as extraction solvent since it was known to produce an extract with appreciable levels of cathinone, cathine and norephedrine from khat [17]. First phase extraction was carried out by spinning the mixture for 15 minutes in Water Bath Shaker (RSB-12) at 120 rpm and room temperature under dark condition. The mixture was filtered using Whatmann filter paper (11 μm pore size), and the residue was re-shaked overnight under the same condition of the former filtrate. Then, the filtrate was concentrated to near dryness using Rota vapor (Heidolph Labo Rota, 4002) at 120 rpm and temperature of 40°C for 1-2 hrs. The residue was measured and dissolved in 20% Tween 80 in physiological saline solution.

Freshness of the khat extract was checked on thin layer chromatography (TLC) following the procedure developed by Lee [18]. Briefly, the plant extract was spotted directly onto a pre-coated *silica gel 60* plate. Cathinone oxalate and cathine oxalate drug standards dissolved in methanol were used as references. The plate was developed in solvent composition of ethyl acetate, methanol and aqueous ammonia with respective proportion of 85:10:5, and viewed under an ultraviolet lamp (254 nm). The spots were visualized using 0.5% ninhydrin solution, and the plate was heated. Cathine appeared purple, while cathinone was a burnt orange moving spot. The R_f_ (retardation factor) values obtained for cathinone and cathine were 0.43 and 0.21, respectively.

#### Acute toxicity testing

Sixteen female Swiss albino mice were randomly divided into 2 groups of 8 mice each. After 2 hrs fasting [19,20], the first and second groups were treated with 500 and 1000 mg/kg of khat extracts, respectively. The animals were checked for two weeks daily for any sign of toxicity such as loss of appetite, hair erection, lacrimation, tremors, convulsions, salivation, diarrhoea, mortality and other signs of overt toxicity [19].

### Mice grouping and treatment

After a one week period of acclimatization in the laboratory, the mice (n = 64) were grouped into eight categories (I-VIII), each containing 8 mice. The first two groups (I-II) were treated with 100 mg/kg of khat extract; the second two groups (III and IV) were treated with 200 mg/kg, while the third two groups (V and VI) were treated with 300 mg/kg of khat extracts daily for 4 weeks following the extract doses used by Girma and Engdawork [21]. The remaining two groups (VII and VIII) were exposed to 0.5 ml of 20% Tween 80 in physiological saline solution for the same duration, frequency and route of administration where one (VII) was used as positive control after infected with *Plasmodium berghei* ANKA (*PbA*) and the other (VIII) as negative control as indicated in the following section.

### Parasite preparation and mice infection

*PbA* parasite was obtained from infected mice, carrying 30% of parasitemia, donated by EPHI. After the mice were terminally anesthetized using diethyl ether and exposure in a closed container, the infected blood was collected through cardiac puncture, and diluted to 1 × 10^5^ PRBCs in PBS. One subgroup from the duplicates of first, second and third groups (I, III, and V) were treated with 100, 200 and 300 mg/kg of khat extract, respectively. The positive control (VII) was infected with 100 μL of the diluted PRBCs (1 × 10^5^) in PBS through intra- peritoneal (i.p.) injection on the last day of the 4^th^ week of the treatment period. Briefly, the experimental animals were divided into groups: (1) neither treated nor infected (n = 8 mice), (2) only treated with khat extract (100, 200 and 300 mg/kg) (n = 24 mice) daily for four weeks but not infected with *PbA*, (3) *PbA* infected, but non khat-treated mice used as positive control (n = 8 mice) and (4) *PbA* infected and khat treated mice (100, 200 and 300 mg/kg) (n = 24 mice) daily for four weeks. Then, the treatment of all mice exposed to methanolic khat extract was extended to day 7 of post infection (7dpi). Starting from 2dpi to the last day of survival of mice (day 7), parasite load, rectal temperature, body weight and Hb levels were monitored.

On the seventh day of post infection, rectal temperature was measured, body weight was taken and few drops of blood samples from tail snip were used for measurement of hemoglobin (Hb) level (Hb analyzer, Hemocue™ haemoglobinometer, Angelholm, Sweden). Parasite load was determined in blood smears, thin and thick, from tail snip stained in 10% Giemsa for 10 min. Percent (%) parasite load was calculated by dividing infected RBCs to total RBCs and multiplying by 100. Parasite loads of nicotine treated mice were compared with the positive control.

### Assessment of severe malaria syndromes

Incidences of clinical signs of cerebral malaria (CM) that involve neurological syndromes were assessed. These were ataxia, paralysis, deviation of the head, convulsions, decrease in body temperature, loss of vascular cell integrity, tissue edema, hemorrhages in the brain of mice, and congestion of micro-vessels with parasitized erythrocytes and/or mononuclear cells [22].

After clinical and physical conditions assessment, each mouse was terminally anesthetized, and blood sample was collected through cardiac puncture for hematological and biochemical tests. Furthermore, some organs such as kidney, liver, spleen, and brain were carefully removed, weighed and processed for histo-pathological study. Lungs were removed and used for assessment of status of pulmonary edema.

### Hematological and biochemical tests

About 0.2 ml of blood samples collected from mice cardiac were used for quantification of total WBCs, lymphocytes, monocytes, RBCs, Haemoglobine (Hb), Hematocrit (HCT), platelets, mean corpuscular volume (MCV), mean corpuscular hemoglobin concentration (MCHC) and mean corpuscular hemoglobin (MCH) using CBC machine (Automated CBC Analyzer: Sysmex KX-21). From 1 ml of the blood sample collected in EDTA coated tube, serum was separated by centrifugation (Centrifuge 4515R) at 10,000 rpm for 10 minutes. The supernatant was transferred into new eppendorf tube and immediately followed by measurement of liver enzymes, serum glutamic oxaloacetate transaminase (sGOT) and serum glutamic pyruric transaminase (sGPT), albumin (Alb), biomarkers of kidney functions such as creatinine (Cr) and urea, inflammation biomarkers; uric acid (UA) and C-reactive protein (CRP) using automated immunochemical analyzer (Axsym MEIA 3rd Generation).

### Pulmonary edema

The status of pulmonary edema was assessed in lungs of mice infected with *PbA* and the positive control. The wet weight of lung was measured immediately after removal of the organ, and the dry weight was determined after overnight incubation at 80°C. Then, the ratio of wet to dry weight was calculated to determine the condition of pulmonary edema [23].

### Histopathological analysis

For histological analysis, liver, kidney and brain of sacrificed mice were collected in 10% buffered neutral formaldehyde; paraffin-embedded brain, liver and kidney tissues were sectioned and stained with hematoxylin and eosin. Slides were coded and scored blind for histological evidence of cerebral syndromes and liver and kidney damages.

### Anti-plasmodial activity of khat extract

#### Mice infection and follow up

For this assay, mice grouping and treatment procedures different from the earlier protocol were used, in that mice infection was commenced after four weeks of oral khat treatment. Accordingly, chloroquine sensitive female Swiss albino mice were grouped into five categories (n = 40), each containing eight mice. Here, all mice were initially infected with 1x10^5^ PRBC in PBS intraperitonally. Then, plant extract was solublized in 20% Tween 80 in physiological saline solution and tested in three doses; 100, 500 and 1000 mg/kg administered to the infected mice (each group contained 8 mice) orally and once daily for four consecutive days. Other two groups: a negative control (n = 8) and a positive control (n = 8) received 20% Tween 80 in saline solution and 10 mg/kg base body weight of chloroquine sulphate, respectively.

#### Suppressive test

Chemo-suppressive activity of the plant crude extract on parasitemia was evaluated following four day suppressive test protocol. Blood sample was taken from tail vein of each mouse on the fourth day p.i. Methanol fixed and 10% Giemsa stained (pH = 7.2 for 10 min) thin film was examined microscopically. Percentage parasitemia and average percentage suppression of parasitemia by the extract were determined following the formula described below [24]:

$$ \mathrm{Mean}\ \%\ \mathrm{parasitemia} = \frac{\mathrm{Total}\ \mathrm{number}\ \mathrm{of}\ \mathrm{pRBCs}}{\mathrm{Total}\ \mathrm{number}\ \mathrm{of}\ \mathrm{RBCs}}\times 100 $$

Percent parasitemia suppression was calculated by using the following formula [25].

$$ \%\ \mathrm{parasitemia}\ \mathrm{suppression} = \frac{\mathrm{Mean}\ \mathrm{parasitemia}\ \mathrm{of}\ \mathrm{control}\ \hbox{--}\ \mathrm{Mean}\ \mathrm{parasitemia}\ \mathrm{of}\ \mathrm{test}}{\mathrm{Mean}\ \mathrm{parasitemia}\ \mathrm{of}\ \mathrm{control}}\times 100 $$

Mean survival time of each mouse in all groups was determined by calculating the average survival days of mice in each group over 30 days. Furthermore, body weight reduction and temperature rise were monitored every 48 hours p.i. using digital balance and thermometer, respectively.

#### Data analysis

Data were checked for their completeness, correctness, and then double entered into Microsoft Office Excel (2007) sheet and analyzed using SPSS version 20.0 software. The analyzed data were expressed in mean ± standard error of mean (SEM), otherwise indicated. One-way analysis of variance (ANOVA) followed by Tukey’s HSD *post*-*hoc* test was employed to compare the effect of crude khat extract on different variables. Mantel-Cox Log-rank test was also used to analyze the survival time between groups. Values of p < 0.05 were considered statistically significant. All assay results were mean of triplicate analysis.

#### Ethical consideration

The study was ethically approved by the Ethical Review Committee of the College of Health Sciences of Jimma University, Ethiopia. Detail experimentation procedures involving mice were managed following the Ethiopian Public Health Institutes (EPHI) animal handling and treatment guidelines.

## Results

### Acute toxicity study

The acute toxicity study made using the current doses of khat extracts did not cause any mortality in all mice within the first 24 h and beyond for period of 14 days. Physical and behavioural observations of the experimental mice also revealed no visible signs of overt toxicity like lacrimation, loss of appetite, tremors, hair erection, salivation and diarrhea.

#### Hematological and biochemical features

According to the result of the *in vivo* study, mice exposed to khat extract followed by *PbA* infection had significantly higher risk of jaundice or liver and renal impairments. This was evidenced by increased level of liver enzymes such as serum GPT and GOT, which were significantly higher (P < 0.001) in blood of mice exposed to the highest dose of khat extract (300 mg/kg) followed by *PbA* infection than the positive control, while albumin level was significantly reduced (P < 0.001). However, creatinine and urea levels did not show any difference between mice treated with khat followed by *PbA* infection and the positive control.

**Figure 1:**
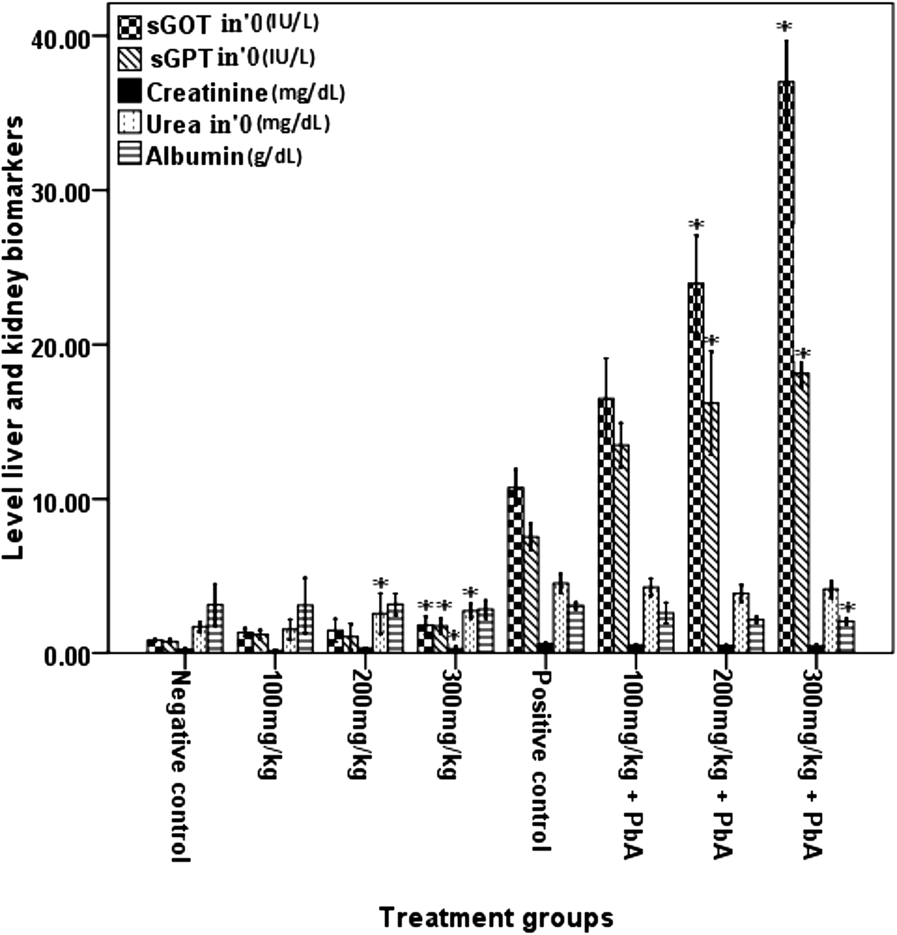
**Levels of liver and kidney biomarkers** **(mean ±** **SEM) of Swiss albino mice (n = 8) treated with khat extracts (100, 200, and 300 mg/kg) daily for 4 weeks, and khat treatment followed by** ***PbA*** **infection [(100, 200 and 300) mg/kg plus** ***PbA*** **infection] and the controls.** Values with asterisk are significantly different (ANOVA, Tukey’s HSD *post*-*hoc* test) from values of the controls.

Influence of khat on the status of liver biomarkers was further observed in mice treated with khat but without *PbA* infection, in a dose dependent manner. Serum GOT and GPT were significantly higher (P < 0.05) in mice treated with higher dose of khat (300 mg/kg) although albumin level did not show significant differences (P > 0.05) between mice treated with khat extract and the negative control. Likewise, in mice treated with higher dose of khat extract (200 and 300 mg/kg) without *PbA* infection, significantly higher (P < 0.05) level of urea was observed than the negative control, while creatinine level was only affected at the highest dose (300 mg/kg) of the khat extract, though it was not detected after *PbA* infection (Figure 1). However, necrosis of kidney and liver tissue were observed in the positive control mice treated with khat followed by *PbA* infection under all doses.

**Figure 2:**
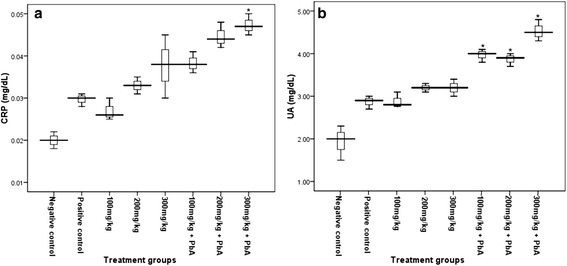
**Level of C-reactive protein (CRP) (a) and uric acid (UA) (b) in Swiss albino mice (n = 8) treated with khat extracts (100, 200, and 300 mg/kg) daily for 4 weeks, and khat treatment followed by** ***PbA*** **infection [(100, 200 and 300) mg/kg plus** ***PbA*** **infection] and the controls.** Values with asterisk are significantly different (ANOVA, Tukey’s HSD *post-hoc* test) from values of the controls.

Inflammation biomarkers such as CRP and UA levels measured in mice treated with khat extract (100, 200 and 300 mg/kg) did not show significant differences (P > 0.05) from the negative control. However, in mice treated with khat extract, under all doses, followed by *PbA* infection, significant increment (P < 0.05) in level of UA than the positive control was observed, while significant rise (P < 0.05) of CRP level was only observed in mice which received 300 mg/kg doses of crude khat extract (Figure 2).

WBCs and Hb levels significantly reduced (P < 0.05) in mice exposed to the highest doses of crude khat extract (300 mg/kg) followed by *PbA* infection compared to the positive controls. In addition, platelets count significantly reduced (P < 0.05) in mice treated with khat extract of the two upper doses of this study (200 and 300 mg/kg), while lymphocyte and RBCs counts were not significantly different (P > 0.05) between the two groups. Effect of khat extract on hematological parameters without *PbA* infection also demonstrated the same pattern as in mice treated with khat extract followed by *PbA* infection. In mice treated with the highest dose of crude khat extract (300 mg/kg), the levels of WBC, Hb, MCV and platelets counts were significantly affected (P < 0.05) (Table 1).

**Table 1:** **Hematological analysis of mice treated with khat followed by** ***Pb*** **A infection with reference to controls (n = 8)**

**Group**	WBC*103/μL	RBC *106/ μL	**Hb (g/dL)**	**HCT (%)**	**MCV (FL)**	**MCH (pg)**	**MCHC g/dL**	Plt*103/ μL	**Lym (%)**
control (−ve)	3.8 ± 0.18	8.07 ± 0.3	12.72 ± 0.47	44.32 ± 1.67	54.85 ± 0.03	15.76 ± 0.05	28.72 ± 0.13	884.25 ± 32.5	87.2 ± 1.86
100 mg/kg^§^	3.52 ± 0.25	8.32 ± 0.29	12.75 ± 0.45	43.42 ± 2.06	52.06 ± 0.64	15.32 ± 0.02	29.46 ± 0.37	810 ± 45.7	80.32 ± 4.23
200 mg/kg^§^	2.85 ± 0.09**	8.7 ± 0.1	12.6 ± 0.18	45.1 ± 0.79	52.9 ± 0.55	15.67 ± 0.3	29.52 ± 0.53	766.7 ± 28.5	85.2 ± 1.72
300 mg/kg^§^	2.03 ± 0.49**	8.33 ± 0.15	11.66 ± 0.1*	43.02 ± 0.7	51.66 ± 0.58*	15.23 ± 0.2	29.42 ± 0.24	770 ± 17.15*	80.06 ± 2.17
Control (+ve)	6.64 ± 0.93	7.9 ± 1.02	14.14 ± 1.74	39.43 ± 5.55	47.8 ± 1.73	17.96 ± 0.9	37.34 ± 1.3	354 ± 68.73	74.92 ± 4.87
100 mg/kg + PbA^§§^	5.14 ± 1.71	6.9 ± 0.72	12.05 ± 1.23	34.47 ± 4.45	48.1 ± 0.89	17.5 ± 1.02	36.2 ± 0.71	328 ± 52	76.12 ± 11.15
200 mg/kg + PbA^§§^	4.57 ± 0.94	7.3 ± 0.34	13.18 ± 0.65	37.06 ± 1.62	50.5 ± 1.23	17.3 ± 1.1	34.5 ± 0.64	173 ± 37.02**	81.85 ± 7.67
300 mg/kg + PbA^§§^	4.09 ± 1.87*	7.1 ± 0.81	11.86 ± 1.48*	37.13 ± 4.43	50.1 ± 0.91	18.06 ± 0.93	36.1 ± 1.02	107 ± 21.66**	77.17 ± 8.09

NB: Plt = platelet, Lym = lymphocyte, *showed significant difference (P < 0.05), **(P < 0.01). Significance level of uninfected but khat treated mice ^§^were compared with –ve control group, while khat treated mice followed by *PbA* infection ^§§^were compared to + ve control group.

**Figure 3:**
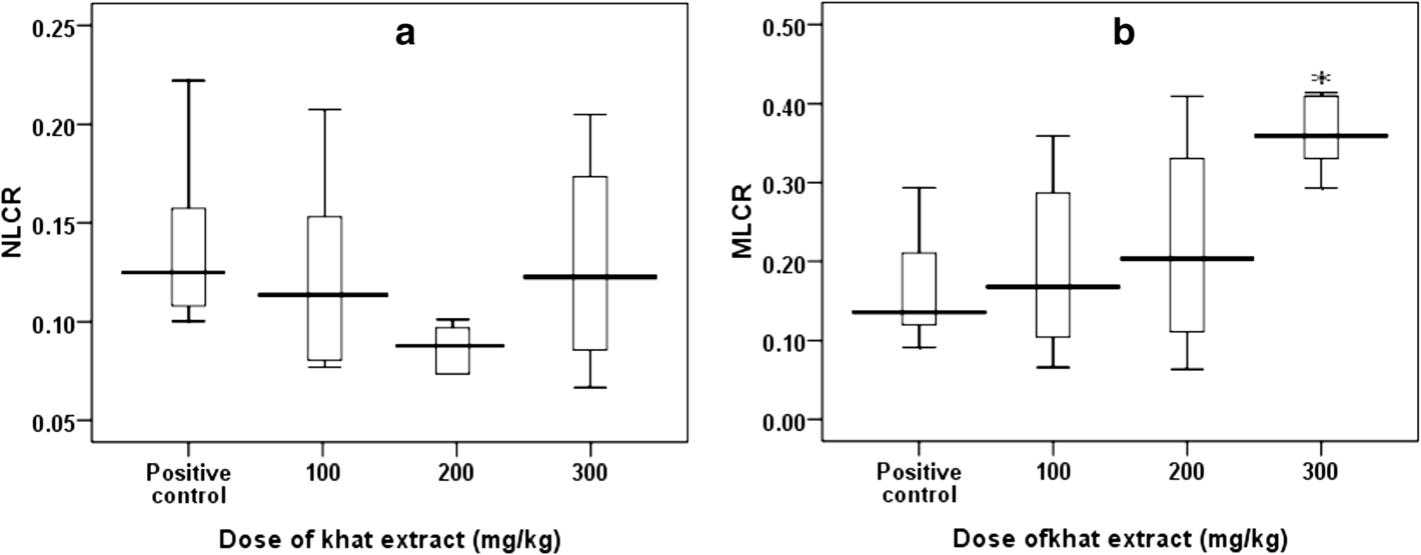
**Box-plots showing distribution of neutorphil-lymphocyte count ratio (NLCR) (a) and monocyte-lymphocyte count ratio (MLCR) (b) of Swiss albino mice treated with crude khat extract (100, 200, and 300 mg/kg) followed by** ***PbA*** **infection.** There was significant difference in the MLCR distribution between the positive control and mice treated with 300 mg/kg dose of khat (One way ANOVA, Tukey’s HSD *post-hoc* test, P = 0.024).

Mice treated with higher dose (300 mg/kg) of crude khat extract showed significant increment (P < 0.05) of monocytes to lymphocyte count ratio (MLCR) compared to the positive control. But, neutrophils to lymphocyte count ratio (NLCR) of the two groups (khat treated and positive control) did not show significant differences (Figure 3).

**Figure 4:**
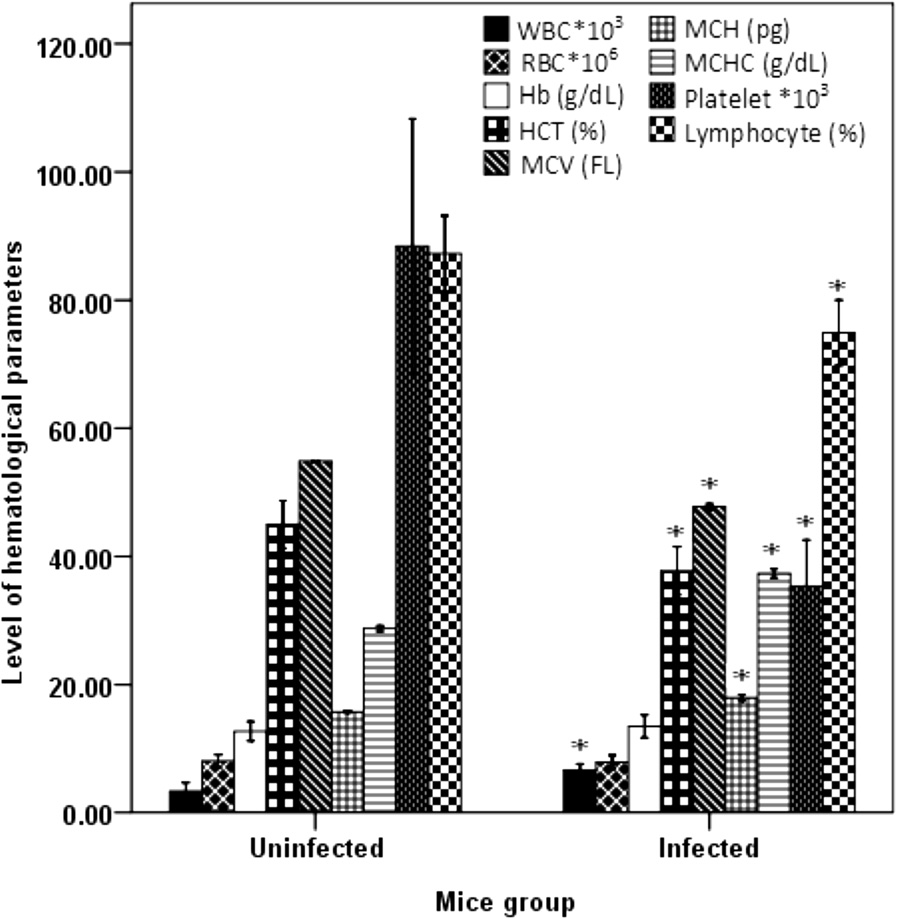
**Hematological parameters (mean ± SEM) of** ***PbA*** **infected and** ***PbA*** **uninfected Swiss albino mice.** Values with asterisk indicate significant difference from values of the uninfected mice (ANOVA, Tukey’s HSD *post-hoc* test).

Assessment made to explore *in vivo* effect of malaria infection on hematological parameters showed that there were significant changes on almost all RBC and WBC indices in *PbA* infected mice. HCT, mean corpuscular volume (MCV), platelet and lymphocyte level were significantly reduced (P < 0.001), while WBC, mean corpuscular hemoglobin (MCH) and mean corpuscular hemoglobin concentration (MCHC) were significantly increased (P < 0.05) in *PbA* infected mice compared to uninfected or negative control (Figure 4).

### Effect of khat on outcome of *PbA* infection

**Figure 5:**
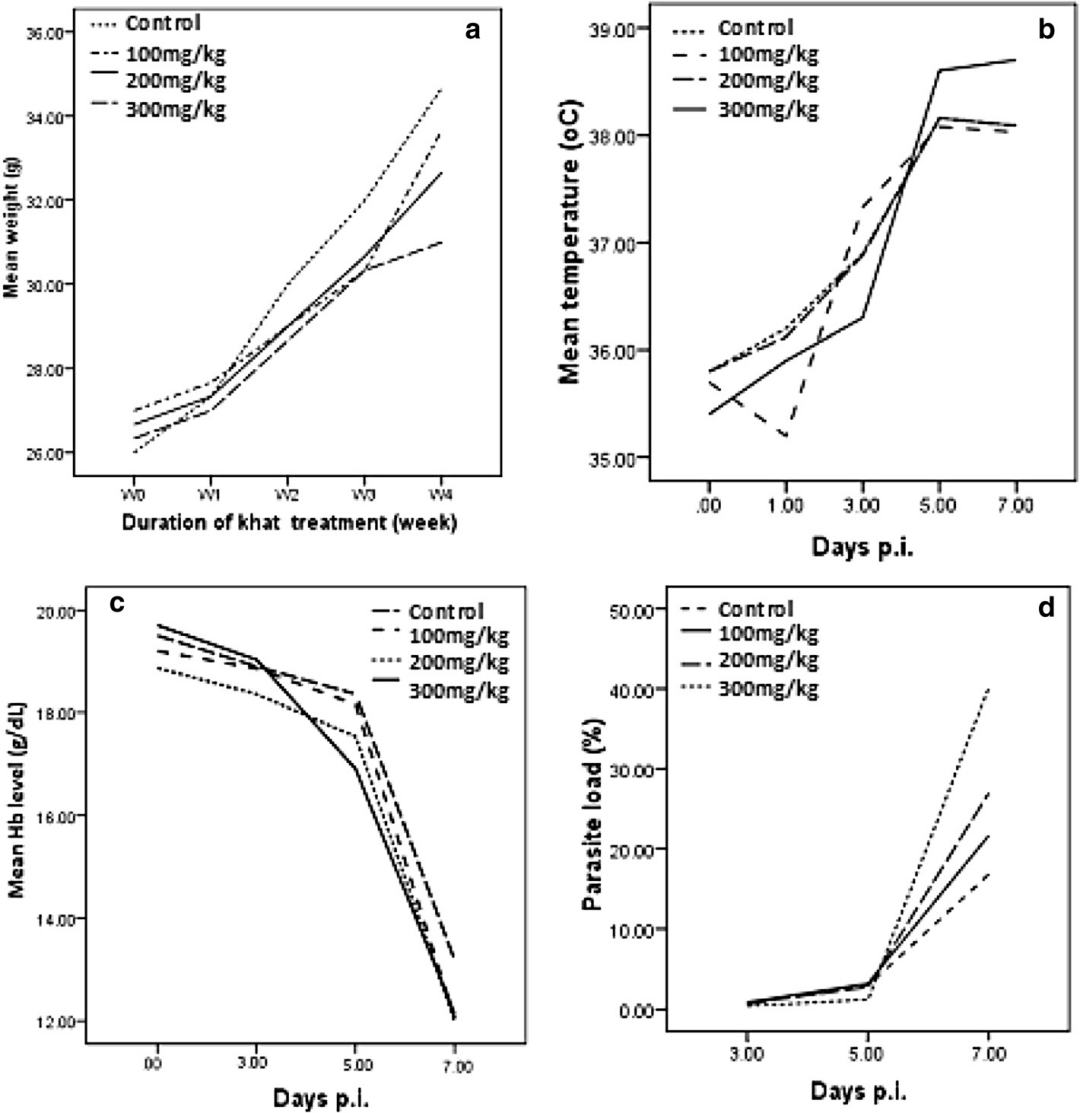
**Body weights of Swiss albino mice treated with khat extract daily for four weeks (a), mean rectal temperature (°C) recorded (b), mean Hb level (g/dL) measured (c), and percentage of parasitemia observed (d) on different days of p.i. of** ***PbA*** **infection.**

Mice exposed to different doses of khat extract (100, 200 and 300 mg/kg) did not show significant difference (P > 0.05) in mean body weight from the control mice during the four weeks of treatment period. Body weight of the animals showed an increment starting from the first to the fourth week of the follow-up period (Figure 5a). Rectal temperature was <36°C for all groups of mice on the day of *PbA* infection (Day 0). Although gradual rising was observed in all groups, in mice treated with 300 mg/kg of khat extract, significant increment (P < 0.01) of rectal temperature was recorded from day 3 to 7p.i. (Figure 5b). Similarly, there was a gradual decline in Hb level in mice treated with khat extract followed by PbA infection. However, a significantly higher reduction (P < 0.05) than the positive control mice was observed after day 5 of p.i. (Figure 5c). Parasite load in mice exposed to different doses of crude khat extract (200 and 300 mg/kg) was significantly lower (P < 0.05) than the control group on the third and fifth day (Figure 5d), but significant increment (P < 0.001) was observed on the seventh day of p.i.

**Figure 6:**
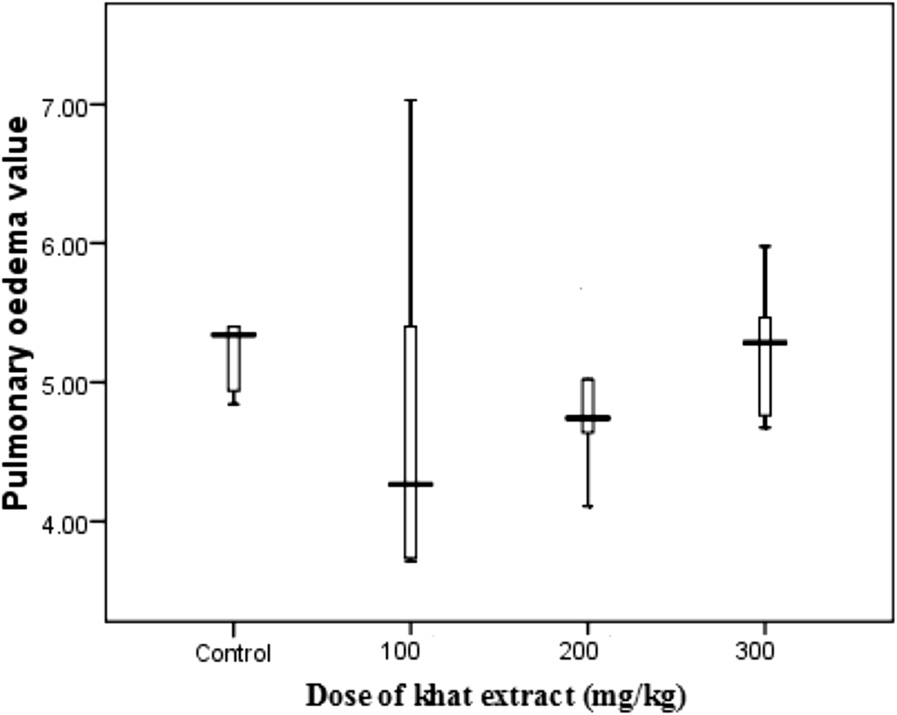
**The status of pulmonary edema (mean ± SEM) of Swiss albino mice (n = 8) treated with crude khat extract followed by** ***PbA*** **infection and positive control.**

Based on the incidence of pulmonary edema assessment, khat extract did not show significant association with increased susceptibility to respiratory problems during *Plasmodium berghei* ANKA infection. This is, there was no significant difference (P > 0.05) between pulmonary edema status in khat exposed mice prior to infection and the positive control (Figure 6).

### Cerebral malaria (CM) state of mice

On the 7^th^ day of p.i. (last day of survival), cerebral malaria symptoms such as limb paralysis, convulsions, shivering, neurological syndromes including ataxia, paralysis, deviation of the head, convulsions, and necrosis of brain tissue observed in all mice irrespective of treatment dose. Due to intense brain necrosis, detection of adherence of parasitized RBC to the endothelial cells was difficult.

### Anti-plasmodial activity of khat

Crude khat extract displayed lower chemo-suppressive activity against *PbA* malaria. Percentage inhibition or suppression analysis showed that under almost all doses of the khat extract (100-1000 mg/kg), the observed percentage parasitemia reduction due to the extract was not significantly different (P > 0.05) from the positive control mice. The percentage suppression observed for all dose of the extract did not exceed 10%. Moreover, maximum survival time of mice infected with *PbA* and treated with khat extract observed was at1000mg/kg, which was only 14 days. This survival time was significantly lower (P < 0.05) than the chloroquine sulphate treated mice (had >30 days survival time). However, the log rank (Mantel-Cox) analysis showed that the overall survival time of mice treated with khat extract was significantly longer (P < 0.001) than the positive control or vehicle mice (Table 2).

**Table 2:** **Parasitemia status, survival time and percentage suppression of parasitemia in infected Swiss albino mice treated with khat extract in the 4 day suppressive test**

**Group**	**Antimalarial activities on day-4 p.i**		**Survival time (day)**
	**% Parasitemia (Mean ± SEM)**	**% suppression/ inhibition**	
Control	21.36 ± 1.32	0.00	8.3 ± 0.11
100 mg/kg	20.7 ± 0.87	3.09	10.8 ± 0.23*
500 mg/kg	19.7 ± 0.65	7.77	11.16 ± 0.68*
1000 mg/kg	19.3 ± 0.22	9.64	13.7 ± 0.52*
CQ (25 mg/kg)	0.00	100	30 ± 0.00

*showed significant difference at P < 0.001, (Kaplan Meier survival analysis, Mantel-Cox Log-rank test) from values of the control.

Rectal temperature measurement made during the *in vivo* anti-plasmodial activity study indicated that mice treated with higher dose of khat extract (500 and 1000 mg/kg) had significantly reduced (P < 0.05) rectal temperature than the control. Also, on the 4^th^ day, the level of hemoglobin and body weight of khat treated mice were comparably reduced in the same manner the positive control mice (Table 3). This indicates that during *PbA* infection in mice, the crude khat extract did not show preventive property against weight loss and anemic condition.

**Table 3:** **Hemoglobin level, rectal temperature, body weight and parasite load of infected Swiss albino mice treated with crude khat extract**

**Group**	**T0 (°C)**	**T4(°C)**	**% change**	**Hb0 (g/dL)**	**Hb4 (g/dL)**	**% change**	**W0 (g)**	**W4 (g)**	**% change**
Control	36.2 ± 0.09	38.16 ± 0.14	−1.96	17.6 ± 0.78	15.3 ± 0.13	2.3	24.8 ± 0.26	22.6 ± 0.33	2.2
100 mg/kg	36.7 ± 0.12	38.2 ± 0.26	−1.5	18.2 ± 0.04	16.3 ± 0.37	1.9	25.4 ± 0.73	23.1 ± 0.41	2.3
500 mg/kg	36.9 ± 0.32	37.5 ± 0.04	−0.6	17.9 ± 0.01	15.1 ± 0.53	2.8	26 ± 1.06	23.8 ± 0.158	2.2
1000 mg/kg	36.5 ± 0.15	37.3 ± 0.22	−0.8	18.5 ± 0.36	15.2 ± 0.25	3.3	25 ± 0.49	23.2 ± 0.14	1.8
CQ (25 mg/kg)	36.7 ± 0.07	36.5 ± 0.13	0.2	18.3 ± 0.44	18.1 ± 0.09	0.2	27 ± 0.37	26.4 ± 0.28	0.6

NB: T0 = rectal temperature on Day 0, T4 = rectal temperature on Day 4, Hb0 = hemoglobin level on Day 0, Hb4 = hemoglobin level on Day 4, W0 = body weight on Day 0, W4 = body weight on Day 4.

## Discussion

Most of the findings of this *in vivo* study were in agreement with our earlier epidemiological study conducted on *P. falciparum* infected khat chewers. Accordingly, a significant change in liver biomarkers in mice treated with khat extract followed by *PbA* infection than the control mice was observed. Change in liver function tests is a hallmark of pathological change in liver during malaria infection [26]. Malaria associated liver dysfunction is usually characterized by a rise in serum bilirubin along with the rise in serum GOT and GPT levels from mild abnormality to more than three times the upper limit of normal [27]. Besides, liver and kidney tissues necrosis observed in a histopathological study further illustrate the possible involvement of these two organs in metabolism and elimination of khat, respectively. Thus, khat use could increase the risks of liver and renal impairment, severe malaria pathologies.

Anemia is a hallmark of malaria infection that occurs as a result of intense hemolysis (destruction) of infected RBCs due to higher parasitemia caused mainly by *P. falciparum* [28]. The remarkable reduction of Hb and HCT level after day 5 of p.i. in mice treated with khat followed by *PbA* infection in this study could be an implication for the relative risk of khat user malaria patients to severe anemia. Thus, the incidence of anemia in mice treated with higher dose of khat was mainly due to hemolysis of infected RBCs as parasite load on 7dpi which was significantly higher. This implies that khat was one of the aggravating factors for incidence of malaria associated anemia among khat users who lack protective activity against hyperparasitemia pathology.

Pulmonary edema, one of the most severe forms of pathophysiology of malaria that involve lung and characterized by increased alveolar capillary permeability leading to intravascular fluid loss into the lungs [29], was not affected by exposure to khat. Usually, this pathology is associated with non-immune individuals with *P. falciparum* infections as part of a severe systemic illness [30]. On the other hand, lower platelet count, a condition known as thrombocytopenia, was manifested in mice chronically exposed to khat prior to the infection irrespective of dose of khat extract. This has a remarkable implication for its possible frequent manifestation among khat user malaria patients. Such reduction in platelet count usually results from apoptosis or activation of platelet [31,32] and their phagocytosis following platelet adherence in the spleen during malaria infection [33,34].

Although WBC counts do not directly involve in malaria infection, it is generally low to normal due to localization of the WBCs from the peripheral circulation to the spleen and other internal organs [35]. Thus, significant reduction in total WBC count observed in mice treated with highest dose of khat (300 mg/kg) followed by *PbA* infection could be attributed to the cytotoxic property of the khat extract [36].

The *PbA* infection in Swiss albino mice was reported to cause neurological problem (the so-called experimental cerebral malaria) [37] and to cause nearly 100% lethality [38]. There is accumulating evidence that the pathogenesis of cerebral malaria is due to an immune-pathological reaction giving rise to excessive production of cytokines such as tumor necrosis factor-alpha (TNF-α) and interferon gamma (IFN-γ) [39]. As all mice treated with khat (100 to 300 mg/kg) prior to *PbA* infection developed neurological symptoms in the same way as the positive control, khat had neither blood brain barrier (BBB) enhancement nor protection potential against cerebral malaria. The elevated level of inflammation biomarkers such as CRP and UA, which triggers secretion of pro-inflammatory cytokines [40], mediators for incidence of cerebral malaria pathology, observed in mice treated with khat extract followed by *PbA* infection strengthen the relative risk of khat users to severe malaria pathologies. Substantial evidences indicated that long term or chronic use of khat increases the level of uric acid [41-45]. Furthermore, the necrosis of brain tissue observed during histopathological study, and clinical manifestations such as ataxia, paralysis, deviation of the head and convulsions in khat treated mice prior to *PbA* infection could strengthen the fact that khat lacks protection against severe malaria pathologies.

In our epidemiological study, the observed reduction in parasite burden among khat chewer malaria patients seems that khat has anti-plasmodial activity. However, following khat treatment extension, the drastic increment of parasite load in mice treated with higher dose of khat extract (200 and 300 mg/kg) followed by *PbA* infection observed certainly question anti-plasmodial activity of the khat. Moreover, the result from anti-plasmodial activity evaluation revealed that except slight reduction on parasite load in khat treated mice, significant parasite suppression was not observed. In support of this, rise of rectal temperature, physical deterioration and severe clinical symptoms appeared in respect to increased parasite load and reduction of Hb level, starting from day 5 p.i. in khat treated mice prior to *PbA* infection. According to Ingyang *et al*. [46], in Swiss albino mice infected with *PbA*, parasitemia started progress on the 2^nd^ day of p.i and reached significant levels from day 4^th^ - 6^th^ and all untreated animals died by the 6^th^ day of infection. Thus, lack of significant effect of khat on some clinical and parasitological state of khat treated mice on early stage of infection cannot be due to absolute suppressive effect of khat, but due to anti-inflammatory activity of the khat [47,48] that can suppress the effect of inflammatory reactions or due to cytotoxicity effect of the plant on the parasite [31]. However, as the parasite load increased and kidney function affected by the parasite and the khat, the inflammation reaction mediated by the uric acid and some parasite’s components [glycosylphosphatidylinositol (GPI)] and toxin released from infected RBC (hemozine) during malaria infection [39] incidence of some pathologies such as cerebral malaria and other could be exacerbated.

Researchers of epidemiological studies reported the effect of malaria infection on hematological parameters differently. Some stated that hematological changes are unreliable laboratory indicators of malaria in acute uncomplicated *P. falciparum* malaria [49], while others reported that lower level of some hematological parameters such as platelet, WBCs, and lymphocyte counts WBCs, eosinophils, RBCs and Hb level when used in combination with other clinical and parasitological method could be the most important predictors of malaria infection [50-53]. Moreover, higher monocytes to lymphocytes count ratio (MLCR) measured in peripheral blood have directly correlates with risk of clinical malaria [54]. This might be due to the major role of the monocytes in the initial stage of innate immune response by releasing cytokines to respond to malaria infection [55]. In agreement to the second notion, our *in vivo* analysis made on association between *PbA* infected mice and haematological parameters showed that almost all, except RBCs count and Hb level, haematological parameters such as WBC, platelet, HCT, MCV, MCH, MCHC and lymphocyte were more significantly reduced in *PbA* infected than in uninfected mice. This finding supports the possible predictor relevance of malaria risks using haematological indicators. Moreover, the high MLCR observed in mice treated with highest dose of crude khat extract (300 mg/kgBW) could be an evidence for increased risk of severe malaria than the positive control [54].

## Conclusions

The finding of this study strengthen an assumption that khat could exacerbate manifestation of most severe malaria symptoms in mice and lacks chemo-suppressive property on *Plasmodium berghei ANKA* parasite.

## Abbreviations

Alb: Albumin ANOVA analysis of variance
BBB: Blood brain barrier
CBC: Complete blood cells count
CM: Cerebral malaria
Cr: Creatinine
EPHI: Ethiopia Public Health institute
HCT: Hematocrit
MLCR: Monocyte-lymphocyte count ratio
NLCR: Neutrophil-lymphocyte count ratio
p.i.: Post infection
PbA: *Plasmodium begrhei* ANKA
pRBC: Parasitized red blood cells
RBC: Red blood cells
Rf: Retardation factor
sGOT: Serum glutamic oxaloacetate transaminase
sGPT: Serum glutamic pyruvic transaminase
WBC: White blood cells
